# Tattooed human in vitro skin model for testing the biocompatibility of tattoo inks and healing progression after tattooing

**DOI:** 10.1038/s41598-025-86813-2

**Published:** 2025-01-17

**Authors:** Kirsten Reddersen, Deborah Maria Gregersen, Jörg Tittelbach, Cornelia Wiegand

**Affiliations:** Department of Dermatology, University Hospital Jena, Am Klinikum 1, 07747 Jena, Germany

**Keywords:** 3D skin model, Tattoo ink, Wound healing, Biocompatibility, Medical research, Cell biology

## Abstract

Tattoos are widespread in the population. Tattoo inks, which contain a variety of ingredients among them hazardous compounds such as polyaromatic hydrocarbons, heavy metals and nanoparticles and that are made for injection into the skin, are not dermatologically tested. New testing systems for evaluation of biocompatibility of tattoo inks as composite products and the tattooing process itself are needed. This paper describes an in vitro 3D human skin model that was tattooed with black and red ink. Biocompatibility including analysis of cytotoxicity, cytokine release, and gene expression patterns of proinflammatory cytokines, proliferation markers, growth factors and structural components was investigated over a period of 7 days. Tattooing of the 3D skin model resulted in a strong inflammatory reaction comparable to in vivo observations that subsided 4 days after treatment. The subsequent healing phase was detectable in the gene expression patterns. Tattooing with two different tattoo inks resulted in distinguishable inflammatory reactions. The described 3D skin model is a useful tool for evaluation of the biocompatibility of tattoo inks and the tattooing process itself and for characterizing the healing process after tattooing.

## Introduction

Tattooing is a very old technique of fixing pigments in the dermal layer of the skin by repeated puncture, which has become more widespread in the last 20 years^1,2^. In 2016, 17.9–21.1% of the German population over 14 years old were tattooed^3^. Tattoo inks contain a variety of ingredients such as insoluble pigments, metals, binders, solvents, emmolient agents, and other additives^4–6^. During tattooing, high amounts of colorants are injected into the skin. A pigment concentration of 2.5 mg/cm^2^skin was quantified in tattooed skin^7^. Tattoo inks have also been found to contain hazardous substances such as polyaromatic hydrocarbons (PAHs), primary aromatic amines (PAAs), heavy metals and nanoparticles^1,4,8,9^. Nanoparticles, which are predominant in black pigments, have been detected not only at the site of tattooing but also in regional lymph nodes and other organs such as the liver^10^. Apart from the ingredients of tattoo inks, potential health hazards associated with tattooing have been identified due to microbial contamination of the inks as well as the abrasion of nickel particles from the tattoo needles as a potential allergen during tattooing^11,12^. Various clinical complications from tattoos like allergenic reactions or infections have been described in the literature^13–15^.

Tattoo inks, which by definition are made for injection into the skin, are not dermatologically tested^13^. Since January 2022, tattoo inks are regulated as chemicals under the REACH system^16^in Europe. Until now, this system has never been applied to composite products, but to pure substances used in the industry. Applying the REACH system, manufacturers of tattoo ink are faced with the problem that more than 4000 chemicals have to be absent or may only be present at very low concentrations in the final product. Apart from this, there is a lack of validated analytical methods for the ink ingredients at the demanded low concentrations^16^.

To improve safety of tattooing, new test systems for evaluation of the biocompatibility of tattoo inks as composite products and the tattooing process itself are needed. At present, no acknowledged in vivo or in vitro model on biocompatibility testing of tattoo inks and wound healing after tattooing is established^17,18^. Existing in-vitro models lack the mechanical injury with destruction of the skin barrier that occurs during tattooing^18^. Standard models to determine potential toxicological risks like carcinogenicity, allergic sensitization potential, or photosensitivity are designed for soluble substances and do not address intradermal application routes. Hence, they are not applicable to tattoo inks or pigments^18^. In the literature, methods described for safety assessment of tattoo inks include animal models like *Daphnia magna*^19,20^, *Xenopus laevis*^20^, murine models^21^, 2D HaCaT cell cultures^19,22^, addition of tattoo inks into the medium^23^or incorporation of pigments as part of the inks into the dermal layer^24^ of 3D skin models. Karregat et al.^25^ described a tattooed 3D skin model for assessment of cytotoxicity and sensitization potential of tattoo inks 24 h after tattooing. However, none of these models assesses the biological characterization of tattooing regarding the cytotoxicity of the inks, the needle trauma for the skin during tattooing itself and the healing process after tattooing.

This paper investigates the toxicity of tattoo inks in vitro and describes the use of a human in vitro 3D full skin model to evaluate effects of tattooing itself as well as with black and red ink. Cytotoxicity, cytokine release, gene expression patterns and histochemical analysis were determined at various time points up to 7 days after tattooing.

## Results

### Cytotoxicity of tattoo ink in 2D keratinocyte models

**Figure 1:**
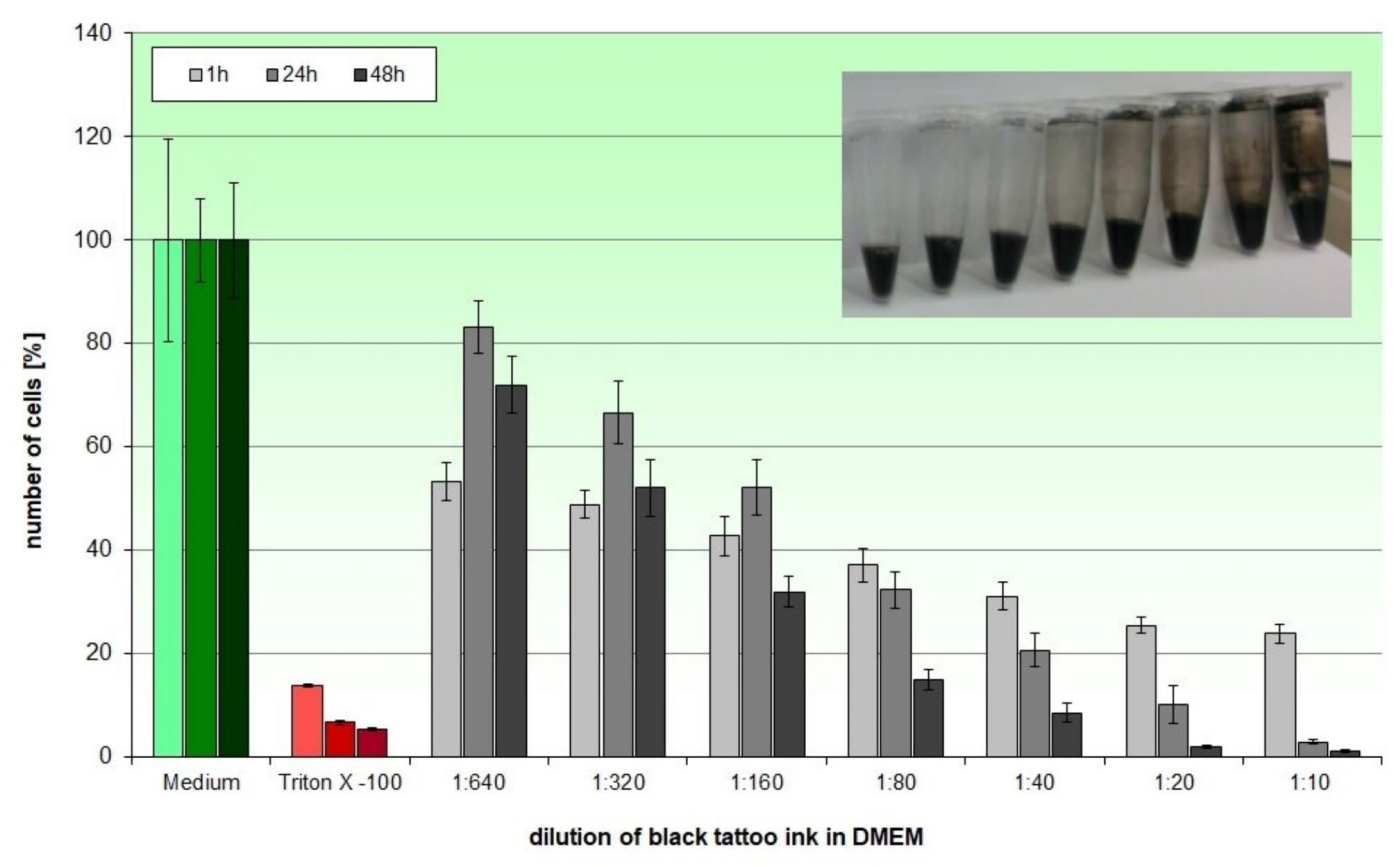
**Fig. 1.** Determination of cytotoxicity of black tattoo ink in 2D HaCaT cell cultures. 1:640 dilution of the ink in medium, which resulted in a still clearly black solution (see embedded graphic) was necessary to obtain a cell compatible concentration after 24 h and 48 h incubation.

Incubation of HaCaT keratinocytes in 96-well plates with diluted black tattoo ink resulted in strong cytotoxic effects on keratinocytes after 1 h, 24 h, and 48 h incubation in this model (Fig. 1). The black tattoo ink had to be diluted up to 1:640 in culture medium to reduce the cytotoxicity of the ink to the point where the cells were sufficiently viable. With this dilution, which is still clearly coloured black (Fig. 1, embedded image), HaCaT keratinocytes showed a viability of around 80% after 24 h incubation.

### Tattooing of 3D skin models

**Figure 2:**
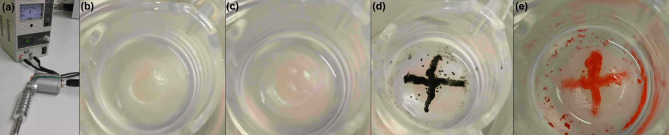
**Fig. 2.** A rotary tattoo machine (a) was used for tattooing 3D skin models (b) without ink (c) or with black (d) or red ink (e).

3D skin models (Fig. 2B) were tattooed with a rotary tattoo machine (Fig. 2A) crosswise without colour (Fig. 2C) and with black or red tattoo ink (Fig. 2D, E). The generated 3D skin models exhibited excellent robustness so that they could withstand the mechanically demanding process of tattooing with repeated insertion of the tattoo needle.

### Histological analysis of tattooed 3D skin models

**Figure 3:**
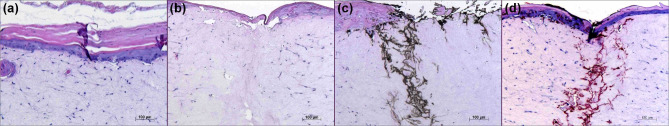
**Fig. 3.** Histology of untreated (a) and tattooed 3D skin models without ink (b) and with black ink (c) or red ink (d). Staining of the models was done with haematoxylin and eosin.

H&E staining of the 3D skin model showed a dermal layer of dark coloured, elongated fibroblasts embedded in collagen. This dermal layer is covered by a layer of differentiated epidermal keratinocytes with a distinct corneocyte layer on top (Fig. 3A). Tattooing without ink results in a clearly visible injury of the epidermal and dermal layer of the skin model (Fig. 3B). At the puncture site, the epidermis is broken and the growth of the fibroblasts in the dermis is clearly reduced or completely disturbed. During the tattooing process, the tattoo needle inserts the ink, which adheres to it into the epidermal and dermal layers with repeated piercing. This leads to the deposition of pigments along the entire puncture channel. Figure 3C and D show the destruction of the epidermis and deposition of ink pigments in the epidermal and dermal compartments of the 3D skin models after tattooing with black and red ink, respectively.

### Determination of cytotoxicity after tattooing of 3D skin models

**Figure 4:**
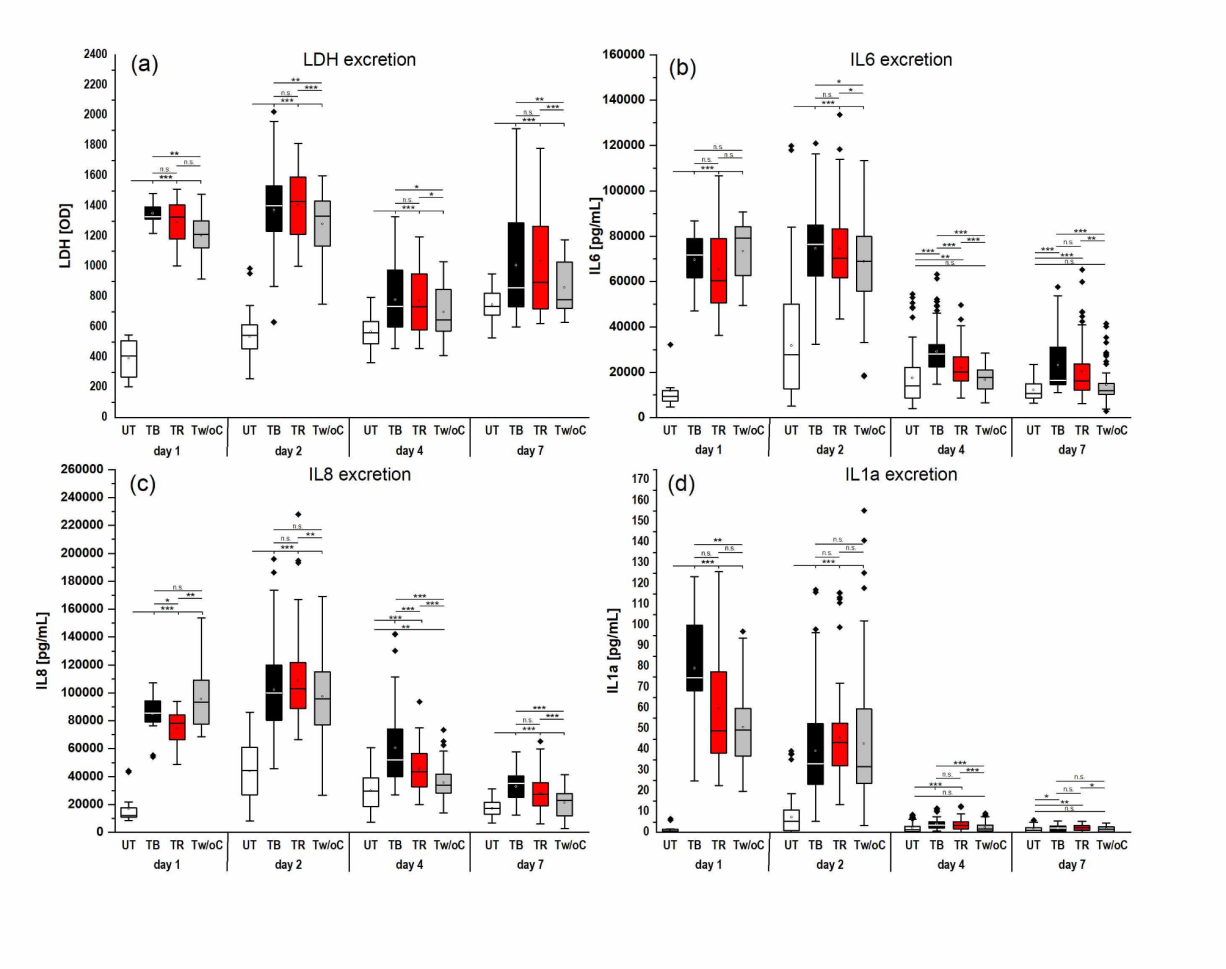
**Fig. 4.** Excretion of the cytotoxic marker LDH (a) and the proinflammatory cytokines IL-6 (b), IL-8 (c), and IL-1α (d) during incubation of 3D skin models after tattooing without and with black or red ink compared to the untreated control during an incubation period of 7 days. Asterisks indicate significant deviations with ∗*P* < 0.05; ∗∗*P* < 0.01; ∗∗∗*P* < 0.001.

Tattooing of the 3D skin models without and with tattoo ink resulted in significantly higher leakage of the cytotoxicity marker LDH into the supernatants, compared to the untreated control (*p* ≤ 0.001). This increase was most evident 24 h and 48 h after tattooing (Fig. 4A). Foremost, the tattooing process caused cell damage. This resulted in high LDH values measured in the supernatants of the models tattooed without ink. 24 h and 48 h after tattooing, tattooed models exhibited thrice the release of LDH of untreated control (Fig. 4A). Introduction of tattoo ink into the skin model further increased cytotoxic effects. At each sampling point, LDH amounts after tattooing with black or red ink were significantly higher compared to models tattooed without ink (*p* ≤ 0.05). There was no significant difference between models tattooed with black or red ink.

### Determination of cytokine levels after tattooing of 3D skin models

Concentration of the pro-inflammatory cytokines IL-6, IL-8, and IL-1α was measured 24 h, 48 h, 96 h, and 168 h after treatment. Tattooing without and with tattoo ink resulted in a strong inflammatory response of the 3D skin models with elevated levels of the cytokines IL-6, IL-8, and IL-1α, with the most noticeable effect after 24 h and 48 h (Fig. 4B, C, D). However, even after 96 h and 168 h, the inflammatory response after tattooing was still visible, e.g. cytokine levels of IL-8 of the tattooed models were significantly higher compared to the untreated control (*p* ≤ 0.01, Fig. 4C). Similar to the induction of LDH release, both mechanical injury and tattoo ink application contributed to the inflammatory process observed.

IL-6 levels of all models tattooed without and with ink were, especially in the first two days, significantly higher compared to the untreated control (*p* ≤ 0.001). At days 2, 4, and 7 after treatment, models tattooed with ink exhibited significantly higher levels of IL-6 compared to the models tattooed without ink (*p* ≤ 0.05, Fig. 4B). Black ink elicited a higher IL-6 release compared to red ink, with differences reaching statistical significance at day 4 (*p* ≤ 0.001).

Similar secretion patterns were observed for IL-8 (Fig. 4C) with significantly elevated levels compared to the untreated control (*p* ≤ 0.001). At days 2, 4, and 7 after treatment, higher IL-8 amounts were detected in the supernatants of the models tattooed with ink compared to models tattooed without ink. As observed for IL-6, concentration of IL-8 was higher for models tattooed with black ink compared to red ink at days 4 (*p* ≤ 0.001) and 7.

IL-1α was released primarily in the first two days after treatment (Fig. 4D). 24 h after treatment, a significant IL-1α increase in tattooed models was observed compared to the untreated control (*p* ≤ 0.001). Again both, mechanical injury and ink application contributed to this effect. Mean IL-1α levels of ink-tattooed models after 24 h were higher compared to models tattooed without ink. Black ink evoked a higher IL-1α release compared to red ink. 48 h after treatment, IL-1α levels of tattooed models continued to be significantly higher compared to the untreated control (*p* ≤ 0.001) with no significant differences regardless of whether ink was used or not. At days 4 and 7, IL-1α levels gradually declined but were still significantly higher in ink-tattooed models compared to the untreated control (*p* ≤ 0.05, Fig. 4D).

### Determination of gene expression levels

**Figure 5:**
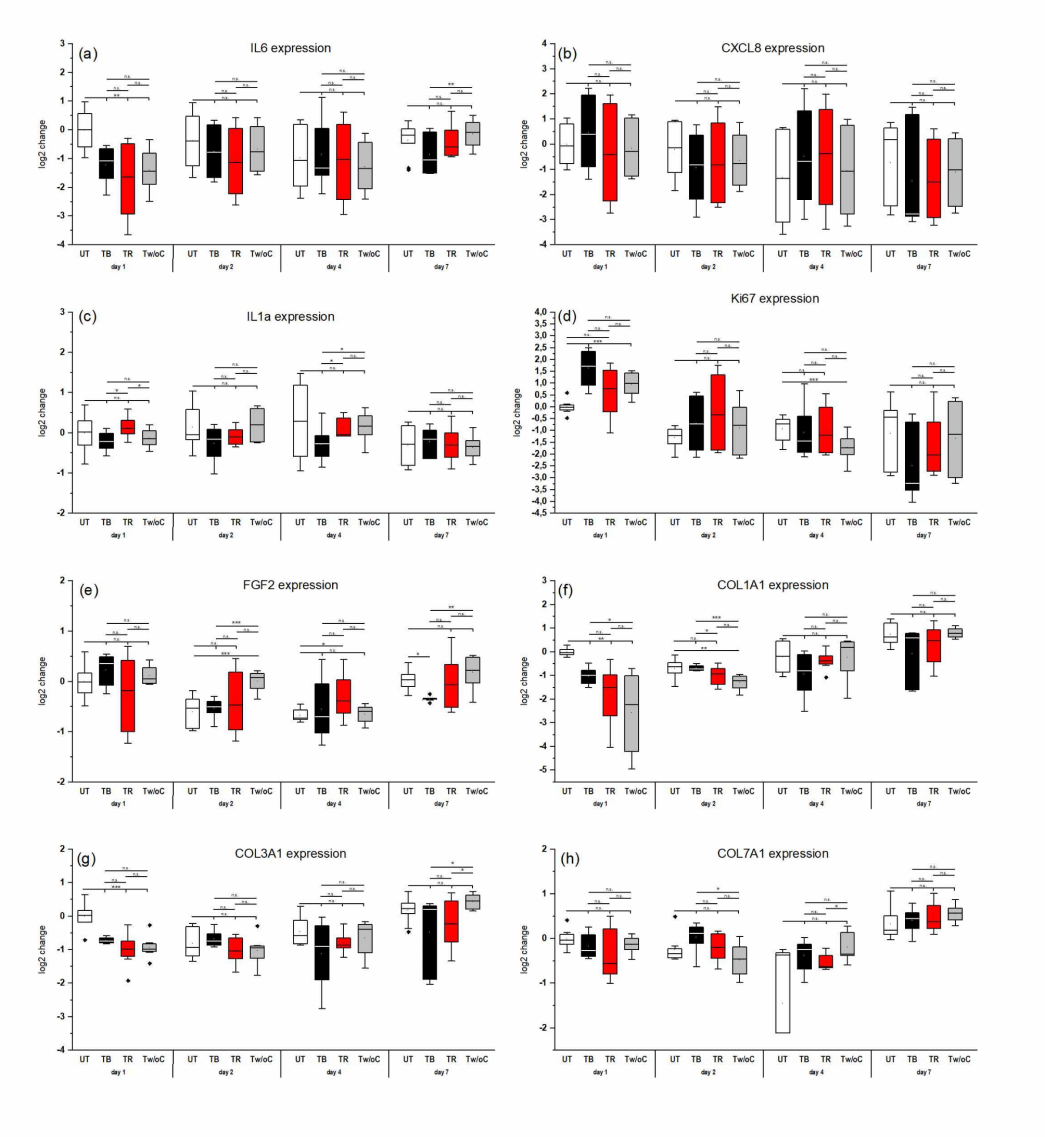
**Fig. 5.** Relative gene expression levels of the proinflammatory cytokines IL-6 (a), IL-8 (b), and IL-1α (c), the proliferation marker Ki67 (d), the growth factor FGF2 (e), and the structural components COL1A1, COL3A1, and COL7A1 during incubation of 3D skin models after tattooing without and with black or red ink compared to the untreated control during an incubation period of 7 days. Transcription levels are expressed as fold changes of the respective untreated control 24 h after treatment. Values were plotted as log2 changes with log_2_ (2) = 1 presenting a doubled upregulation and log_2_ (0,5)=−1 presenting a downregulation by factor 2 compared to the control. Asterisks indicate significant deviations with ∗*P* < 0.05; ∗∗*P* < 0.01; ∗∗∗*P* < 0.001.

Expression of mRNA levels of different genes associated with proinflammatory processes, proliferation, and regeneration during healing were analysed at days 1, 2, 4, and 7 to evaluate the cellular responses of the 3D skin models to tattooing. This included the gene expression of inflammatory mediators (IL6, CXCL8, and IL1A), for proliferation processes (KI67), growth factor (FGF2), and structural components (COL1A1, COL3A1, and COL7A1). The gene expression of the pro-inflammatory mediators IL6, CXCL8, and IL1A was decreased compared to the control over time (Fig. 5A, B, C). This downregulation was most evident for expression of IL6 24 h after tattooing (Fig. 5A). No statistically significant differences were observed for tattooing with or without ink. Gene expression of the proliferation marker KI67 was upregulated 24 h after tattooing. This effect was observed for tattooing without ink (*p* ≤ 0.001), but was even higher after tattooing with ink and achieved the highest effect with black ink (*p* ≤ 0.001, Fig. 5D). At day 2, this effect was still observable, but to a much lesser extent. At days 4 and 7, gene expression of KI67 was downregulated for tattooed models compared to the control. The gene expression of the growth factor FGF2 was upregulated for tattooed models compared to the untreated control, most evident at day 2 and 7 after treatment (Fig. 5E). This effect of upregulation of the fibroblast growth factor after wounding was reduced when ink, especially black ink, was applied. Regeneration of the dermal layer after tattooing was observed by the gene expression of COL1A1, COL3A1, and COL7A1. At day 4 and 7 after tattooing, for all three genes an upregulation was observed for tattooed models compared to the untreated control. This upregulation of collagen expression was most evident for models tattooed without ink. Application of ink, especially the black ink, reduced the regeneration process of the dermal layer after wounding (Fig. 5F, G, H).

## Discussion

In our studies, cytotoxicity testing of black tattoo ink using 2D HaCaT cell cultures revealed the limitations of 2D cell cultures. The black ink had to be diluted 1:640 in medium to achieve a cell compatible concentration, which is far from the application reality during tattooing. Arl et al. also tested the cytotoxicity of different tattoo inks in HaCaT 2D cell cultures^19^. Cell compatible concentrations of the tested inks ranged from 1:1000 to 1:10000 dilution, depending on the ink, which is comparable to our results. These systems display a much higher sensitivity compared to the in vivo situation^26^, which could lead to an over interpretation of negative effects and too little selectivity with regard to safety aspects. Another major problem in the analysis of tattoo inks in standard analytical assays arises from the fact, that these inks contain insoluble pigments. These can be inaccessible during analysis or may complicate the analytical procedure due to assay interactions. This was observed in our study for testing red ink in 2D cell culture (data not shown). The problem of differences in solubility of tattoo ink ingredients during risk assessment was also addressed by the EDQM^17^. To overcome the analytical limitations observed in 2D cell cultures and to simulate the in vivo situation as closely as possible as it occurs during tattooing, we have successfully employed 3D skin models for testing local tattooing effects. In these models, pigments of the tattoo inks remained in the 3D skin models and were not observed in the supernatants, so that analytical interferences are not to be expected. Using a rotary tattoo machine, the models were pricked with a rate of about 5,000 x per minute^13^, which is a highly challenging mechanical process. The models exhibited excellent robustness during the tattooing process without detachment of the epidermis. Histochemical analysis of the models tattooed with black and red ink showed pigment distribution along the whole puncture channel involving the epidermis and dermis of the models.

As expected, a strong cytotoxic effect and inflammatory reaction was elicited in the 3D skin models after tattooing. Increased levels of the cytotoxic marker LDH were noticed during the entire observation period. LDH is released from the cytosol after cell membrane damage during necrotic cell death into the medium^27^. Proinflammatory cytokines like IL-6, IL-8 and IL-1α, small soluble proteins secreted from keratinocytes and fibroblasts, act as part of a complex signal cascade for regulation of acute and chronic inflammation. IL-6 plays an important role in wound healing as it exerts a mitogenic effect on keratinocytes^28^. IL-8 as a member of the CXC chemokine subfamily plays a role in chemotactic recruitment of neutrophils to the wound site and has proliferative effects on keratinocytes^28^. IL-1α is constitutively stored in a number of cells e.g. keratinocytes and fibroblasts as pro-IL-1α^29^. IL-1α is designated as key ´alarmin´ in the cells that alerts the host to injury and activates the inflammation cascade^29^. In accordance, tattooed and ink-tattooed 3D skin models exhibited elevated secretion of the proinflammatory mediators. Karregat et al. observed comparable results when analysing the cytotoxicity and release of IL-8 and IL-1α 24 h after injection of different tattoo inks into a skin model using a permanent makeup machine^25^. Cell viability was significantly reduced and IL-8 as well as IL-1α levels were significantly higher compared to the untreated control, with levels depending on the respective ink. Bil et al.^23^ tested cytotoxicity and sensitization potential of 5 inks by adding the diluted ink into the culture medium of 3D skin models for 24 h. The inks had to be diluted up to 1:10 000 to achieve a cell compatible concentration in the medium. Hering et al.^24^ described a model for testing tattoo pigments in 3D skin models with incorporated pigments in the dermal compartment. The authors didn´t observe increased secretion of IL-6 and IL-8 of the pigmented models compared to the untreated control. However, their results are not comparable to our results. The model described by Hering et al. simulates the healed tattoo with pigments in the dermis without wounding and additional ingredients of tattoo inks. Additionally, supernatants for analysis of interleukins were changed prior to testing, so release of cytokines from the cells that were in contact with the pigments in the dermis for 2–3 weeks before analysis were not detectable.

The results of our study indicate that the cytokine release observed is triggered by mechanical damage due to pricking in combination with the toxic effects of the tattoo ink on the human cells in the model. It was found that the release of the cytotoxic marker LDH and the cytokines interleukin-6, interleukin-8, and interleukin-1α of ink-tattooed models was higher at different time points compared to models tattooed without ink. Karregat et al.^25^ also observed an increase in IL-1α release of ink-tattooed models compared to vehicle-tattooed models 24 h after treatment while IL-8 release was not affected. However, our study showed that although differences in IL-8 release were not detectable 24 h after treatment, they became clearly noticeable at days 2, 4, and 7 after treatment, highlighting the need for longer observation periods.

We could further show that models tattooed with black ink exhibited higher levels of proinflammatory marker compared to models tattooed with red ink. Black tattoo ink contains nano-scaled carbon particles and may contain carcinogenic compounds like PAHs^1,30]^. Because of their high surface-to-volume ratio, nanoparticles are very reactive and have an extremely high bioavailability of toxic or carcinogenic compounds^1,31]^. Hence, the composition of the black ink used in our study might explain the observed higher proinflammatory effects compared to the red ink.

In our model, the highest increase of the cytotoxic and proinflammatory markers was observed 24 and 48 h after tattooing. The inflammatory reaction subsided after 4 and 7 days with increased healing of the 3D skin model, noticeable as a decrease in IL-6, IL-8, and IL-1α release. Similar processes are described in literature after tattooing people. Maeda et al.^32^ investigated inflammatory reactions at different time points after tattooing. They observed an acute inflammation 90 min after injection of tattoo ink that persisted until the sixth day. Sperry et al.^33^ made similar observations of a rapid onset of an inflammatory reaction after tattooing that is resolved 7–10 days after skin puncture.

In our study, healing of the skin models after tattooing was investigated by measurement of gene expression levels of KI67 as proliferation marker, FGF2 as growth factor and collagens as structural components. Tattooing resulted in a strong increase of the gene expression of Ki67. Ki67 is present during all active phases of the cell cycle and hence makes an excellent marker to observe the growth fraction of a cell population^34^. Regeneration of the 3D model after tattooing was also observable in the upregulation of FGF2 gene expression as well as the structural components of the dermal compartment like the genes COL1A1, COL3A1, and COL7A1. In wound healing, matrix remodelling following the inflammatory phase is characterized by collagen synthesis, which was also described for 3D skin models 5 days after microneedling^35^. This is the first time, similar observations can be reported for the tattoo process. However, the healing process was distinctly inhibited in models tattooed with ink, especially black ink, highlighting the toxic effects of currently widely used tattoo inks.

Compared to the literature on inflammatory reactions and healing processes after tattooing in humans, the model described by us is very well suited to simulate these reactions and to distinguish the biological effects of different tattoo inks after tattooing. Hence, it is a useful tool for evaluation of the biocompatibility of tattoo inks and the tattooing process itself and for characterizing the healing process after tattooing.

## Materials and methods

### Cytotoxicity of tattoo ink in 2D keratinocyte models

Cytotoxicity of tattoo ink in 2D keratinocyte models was determined as described elsewhere^36^. In brief, HaCaT keratinocytes (a gift of Prof. Dr. N.E. Fusenig, DKFZ, Germany) were incubated in 96-well microtiter plates with black tattoo ink (alpha SUPERFLUID 90% Black, Deep Colours GmbH, Germany) diluted 1:10, 1:20, 1:40, 1:80, 1:160, 1:320, and 1:640 in Dulbecco’s modified Eagle medium (DMEM, Promocell, Germany) for 1 h, 24 h, and 48 h. Viability of the HaCaT keratinocytes was determined using the luminometric ATPLite Kit (Perkin Elmer Life Sciences, US).

### Generation of 3D skin models

3D skin models were generated according to Reddersen et al.^26^. Briefly, fibroblasts at a density of 1 × 10E5/mL were mixed with rat-tail collagen and cultivated submerse for 7 days. At day 8, keratinocytes were seeded, cultivated submersed for 10 day followed by 10 days airlift cultivation. Quality of matured 3D skin models was controlled by histological analysis using haematoxylin/eosin staining.

### Tattooing of 3D skin models

Mature 3D skin models were tattooed crosswise with 1205RL needles (INKgrafiX^®^, Germany) with a needle depth of 1 mm under sterile conditions using the IG-7 g rotary tattoo machine with a voltage of 4.8 volts (INKgrafiX^®^, Germany). Models were tattooed with undiluted black ink (alpha SUPERFLUID 90% Black, Deep Colours GmbH, Germany), or undiluted red ink (Feuerrot Sailor Jerry, Deep Colours GmbH, Germany). The black ink contains according to the label aqua, ammonium acrylates copolymer, CI 77,266, hamamelis virginiana extract, ethylpropandiol, glycerine, PEG-8, caprylyl glycol, simethicone, phenylpropanol, and benzoic acid. Main ingredients of the red ink as labelled are aqua, alcohol, glycerine, CI 56,110, rosa damascene extract, and methyl ethyl ketone. Models tattooed without ink and untreated 3D skin models served as controls. Samples of 3D skin models and 4 mL of supernatants were taken 24 h, 48 h, 96 h, and 168 h after tattooing and kept frozen at −20 °C until analysis. Medium was refreshed every 2 days after treatment.

### Histological analysis

Samples were fixed in formalin solution (4%, Hollborn, Germany) followed by embedding in paraffin blocks (Merck, Germany) using standard histology protocols. 4 μm thick sections were stained with hematoxylin and eosin (H&E, Merck, Germany) and imaged and photographed using the Axio Scope A.1 (Carl Zeiss AG, Germany) coupled to the AxioCam MRc camera (Carl Zeiss AG, Germany) and analysed using AxioVision 4.9 software (Carl Zeiss AG, Germany).

### Determination of cytotoxicity

Cytotoxicity was analysed by measuring the activity of lactate dehydrogenase in the supernatants of the 3D skin models at each sampling point using a cytotoxicity detection kit (Roche, Switzerland)^26^.

### Determination of cytokine levels using ELISA

Cytokine levels of interleukin 6 (IL-6), interleukin 8 (IL-8), and interleukin 1α (IL-1α) were determined in the supernatants using human interleukin (IL)−6 (Mabtech, Sweden), IL-8 and IL-1 α (R&D Systems, USA) enzyme-linked immunosorbent assay kits according to the manufacturers’ instructions as described by Reddersen et al.^26^. 100 µL of the supernatants of the 3D skin models were analysed in duplicate for each cytokine.

### Determination of gene expression levels using real time PCR

RNA of the 3D skin models was isolated and analysed using real time polymerase chain reaction (PCR)^26^. Briefly, RNA of the completely homogenized 3D skin model was isolated using the Qiagen RNeasy Mini Purification Kit (Qiagen, Germany). After conversion of 20 ng isolated RNA to cDNA using the High Capacity cDNA Reverse Transcription Kit (Life Technologies, USA) real-time polymerase chain reaction (PCR) was carried out using the KAPA SYBR FAST qPCR Kit (KAPABiosystems, USA). Transcription levels are expressed as fold changes of the respective untreated control 24 h after treatment. Values were plotted as log2 changes with log_2_ (2) = 1 presenting a doubled upregulation and log_2_ (0,5)=−1 presenting a downregulation by factor 2 compared to the control. Table 1 lists primer sequences used in these experiments.

**Table 1:** Primer sequences used for real time PCR.

Gene name	Primer sequence (5´->3´)	
	Forward primer	Reverse primer
interleukin 6	CCA CCG GGA ACG AAA GAG AA	GAG AAG GCA ACT GGA CCG AA
interleukin 8	ATG ACT TCC AAG CTG GCC GT	TCC TTG GCA AAA CTG CAC CT
interleukin 1α	CGC CAA TGA CTC AGA GGA AGA	AGG GCG TCA TTC AGG ATG AA
COL7As	TTG TGG TGT CAG ATG CAA CG	TGT GAT GTC TGT GGC AGT AGA G
Ki67	QT00014203 (Quiagen)	
FGF2	QT00047579 (Quiagen)	
COL1A1	QT00037793 (Quiagen)	
COL3A1	QT00058233 (Quiagen)	
β-actin (housekeeper)	QT01680476 (Quiagen)	

### Statistics

Experiments were performed twice with two technical replicates each, so each sample was measured in four replicates. Evaluation was performed using Microsoft^®^ Excel 2016 and OriginPro2019 (OriginLab Corporation). All values presented are expressed as means ± SD. One-way analysis of variance was carried out to determine statistical significances (Microsoft^®^ Excel 2016). Differences were considered statstically signifiant at a level of *p* ≤ 0.05 (*), *p* ≤ 0.01 (**) and *p* ≤ 0.001 (***).

## Data Availability

All outcome data are available as representative images in the main text. The raw datasets generated and analysed during the current study are available from the corresponding author on reasonable request.
